# Advances in Design Strategies of Multiplex Electrochemical Aptasensors

**DOI:** 10.3390/s22010161

**Published:** 2021-12-27

**Authors:** Iwona Grabowska, Maria Hepel, Katarzyna Kurzątkowska-Adaszyńska

**Affiliations:** 1 Institute of Animal Reproduction and Food Research, Polish Academy of Sciences, Tuwima 10, 10-748 Olsztyn, Poland; 2 Department of Chemistry, State University of New York at Potsdam, Potsdam, NY 13676, USA; hepelmr@podstam.edu

**Keywords:** electrochemical methods, aptamers, aptasensors, simultaneous detection, multi-label approaches

## Abstract

In recent years, the need for simple, fast, and economical detection of food and environmental contaminants, and the necessity to monitor biomarkers of different diseases have considerably accelerated the development of biosensor technology. However, designing biosensors capable of simultaneous determination of two or more analytes in a single measurement, for example on a single working electrode in single solution, is still a great challenge. On the other hand, such analysis offers many advantages compared to single analyte tests, such as cost per test, labor, throughput, and convenience. Because of the high sensitivity and scalability of the electrochemical detection systems on the one hand and the specificity of aptamers on the other, the electrochemical aptasensors are considered to be highly effective devices for simultaneous detection of multiple-target analytes. In this review, we describe and evaluate multi-label approaches based on (1) metal quantum dots and metal ions, (2) redox labels, and (3) enzyme labels. We focus on recently developed strategies for multiplex sensing using electrochemical aptasensors. Furthermore, we emphasize the use of different nanomaterials in the construction of these aptasensors. Based on examples from the existing literature, we highlight recent applications of multiplexed detection platforms in clinical diagnostics, food control, and environmental monitoring. Finally, we discuss the advantages and disadvantages of the aptasensors developed so far, and debate possible challenges and prospects.

## 1. Introduction

Since the first two independent reports were published in 1990 on the in vitro selection and amplification method for RNA sequences (then termed aptamers) able to bind specific ligands [1,2], aptamers have gained tremendous interest from researchers around the world. Both RNA and, discovered two years later, DNA aptamer sequences [3] offer many unique properties, including specificity, high affinity, and respectable chemical, thermal, and conformational stability. These analogs of antibodies can readily undergo reversible folding upon binding with target molecules [4]. The respective oligonucleotide sequences can be obtained in vitro using a Systematic Evolution of Ligands by the EXpotential enrichment (SELEX) process. So far, a number of ssDNA and ssRNA sequences have been identified that bind to a broad range of target analytes, e.g., proteins [5], peptides [6], amino acids [7], antibiotics [8], vitamins and minerals [9], metal ions [10], and even whole cells [11], or microorganisms such as bacteria [12] or viruses [13]. Moreover, because of the easy and controllable chemical modification of ssDNA and ssRNA sequences and their low cross-reactivity, these new artificial biological receptors have become an attractive alternative to antibody-based immunosensors [14,15,16,17].

In recent years, progress has been observed in the relevant research in the area of aptasensors. Many aptamer-based biosensors with electrochemical transduction mechanisms have been proposed, owing to their considerable advantages over other types of aptasensors. These advantages include high sensitivity, selectivity, scalability, simplicity of accompanying measuring devices, reliability, convenient handling, and suitability for in-field and point-of-care medical diagnostics [18]. In our opinion, further development in this field will be related the application of new specific aptamers, which because of the SELEX method could possibly be selected for any kind of molecule.

Numerous electrochemical aptasensors have been developed so far for the detection of single analytes that are important from the points of view of (1) disease diagnostics [19,20,21,22,23,24], (2) food analysis [25,26,27], and (3) environmental monitoring [28,29]. Moreover, several review papers discussing current achievements and future perspectives in the area of electrochemical aptasensors have recently been presented [30,31,32]. Only a few examples are cited here. In addition, during the last decade, different advanced functional materials and novel nanostructures have been extensively explored for use in the construction of electrochemical aptasensors [33,34,35]. Nanomaterials, such as 2D and 3D carbon nanoforms, and metal, semiconductor, magnetic, and up-conversion nanoparticles, including quantum dots, or black phosphorus-based crystals, have been successfully implemented in electrochemical aptasensor construction owing to their excellent electronic, optical, mechanical, and thermal properties [36,37].

The attention of scientists toward the electrochemical multiplex detection of several analytes in a single run has recently been growing steadily. However, the majority of the papers still report on the use of antibodies as biological receptors in biosensing platforms [38,39,40,41,42].

**Scheme 1:**
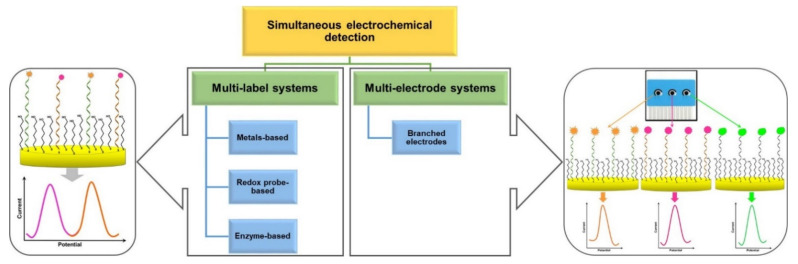
Sensing modes of multi-label and multi-electrode electrochemical aptasensors for simultaneous detection of multiple analytes.

In general, simultaneous electrochemical detection of analytes could be achieved by either multi-label or multi-electrode approaches, as shown in Scheme 1. The idea of multi-label approaches is based on a single electrode that is able to produce distinguishable electrochemical signals enabling the detection of the corresponding compounds. Metal ions, quantum dots, redox agents, redox active polymers, and enzymes have been employed as electrochemical labels in this approach [43]. On the other hand, multi-electrode platforms consist of multiple sensing areas, which are able to detect particular analytes [44,45]. They can be divided into branched (sub-grouped as chip-based and disposable electrodes) and paper-based electrodes. The most desirable features of multi-analyte assays are the low cost per test, low labor intensity, high throughput, and operational convenience.

Simultaneous detection of multiple analytes is extremely important, especially for the biomarkers of different diseases, such as cancer, cardiovascular diseases, diabetes, or others. It can help in the early screening of the disease and the monitoring of its therapeutic response. Furthermore, the detection of multiple biomarkers can help predict the severity of the disease, its progression, and its mortality rate [46]. Moreover, the detection of a single component has frequently been insufficient, as for example in food safety monitoring, since many toxic compounds can co-exist animal feed and human food, such as in the case of mycotoxins, aflatoxin B1 (AFB1), and ochratoxin A (OTA). This co-existence could even enhance their toxicity because of additional or synergistic effects [47].

In this review, we present a comprehensive overview of the multiplex electrochemical aptasensors (MEAS) designed for simultaneous detection of several analytes. We begin with a discussion of the general principles of electrochemical aptasensors based on the nanoparticles of metal compounds, such as the metal chalcogenide quantum dots (PbS, CdS, ZnS, and CdTe) and perovskites (LaMO_3_), followed by metal ions (Pb^2+^, Cd^2+^, and Cu^2+^), metal ion redox labels, and the nanomaterials used for their encapsulation or immobilization. Subsequently, the organic redox probe-based electrochemical aptasensors are reviewed to delineate various types of redox labels, such as ferrocene, antraquionone, thionine, methylene blue, and hemin. Then, we focus on the enzyme-based electrochemical aptasensors and analyze different mechanisms used for analytical signal generation and its amplification. The use of modern nanomaterials in all of these electrochemical aptasensors is highlighted. Finally, in the last section, the challenges and future perspectives of designing electrochemical aptasensors for multiple analytes detection are presented.

## 2. Multiplex Electrochemical Aptasensors Based on Quantum Dots and Metal Ions

**Scheme 2:**
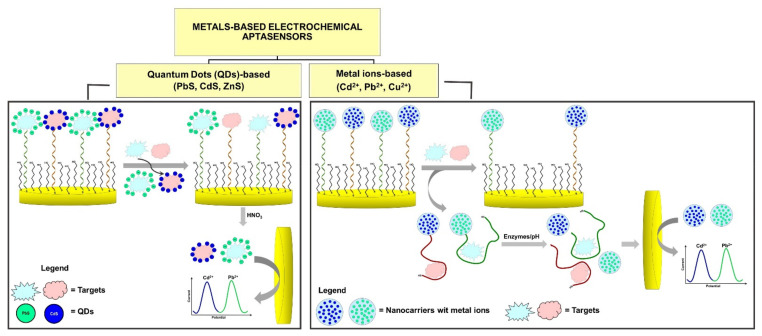
General sensing mechanisms of multiplex electrochemical aptasensors (MEAS) based on quantum dots, metal chalcogenide NPs, and heavy metal ions.

Heavy-metal chalcogenide quantum dots (QDs) and metal ions have been widely used in electrochemical aptasensors as the redox active labels for simultaneous detection of multiple analytes. The general principles of the sensing mechanisms of these sensors are illustrated in Scheme 2.

The multiplex electrochemical aptasensors based on quantum dots, metal compound semiconductor NPs, and metal ions, developed for various groups of analytes, are listed in Table 1.

It can be seen that among the QDs, PbS and CdS dominate [48,49,50,52,60], as well as ZnS [54], CdTe [58], and QDs encapsulated in nanomaterials, such as the metal-organic frameworks PbS@ZIF-8 and CdS@ZIF-8 [63]. On the other hand, various composite and nanostructured materials were mainly loaded with Cd^2+^ and Pb^2+^ or Cu^2+^ cations, and included nanospherical branched polyethylene imine brushes (MSPEIs) [51], an amine functionalized nanoscale porous metal-organic framework (NMOF) [53,56,57], magnetic hollow porous nanoparticles (MHPs) [55], apoferritin [64], Au/BSA nanospheres [59], and polydopamine-LaMO_3_ perovskite (PDA-LMO) [65].

The concept of simultaneously detecting two proteins, thrombin and lysozyme, based on coupling specific aptamers with QD semiconductor nanocrystals, CdS and PbS, was demonstrated for the first time by Hansen and co-workers [48]. The idea of this assay relies on a single-step displacement of QD-protein conjugates (CdS-thrombin and PbS-lysozyme bound to aptamers immobilized on a Au substrate) by target proteins (thrombin and lysozyme) present in the sample analyzed. The released protein labelled with CdS and PbS nanocrystals was dissolved by 0.1 M HNO_3_. In the next step, the protein targets were identified in a concentration-depended manner by the corresponding metal peaks detected electrochemically in square-wave stripping voltammetry. Owing to the large amplification caused by the presence of numerous nanocrystals with every tagged protein molecule, a very low detection limit, in the range of attomolar concentrations, was attained [48]. The principle of subsequent assays applying PbS and CdS nanoparticles was shown by Li and co-workers [49]. In this case, a dual aptamer sequence, containing units specific for adenosine and thrombin, and the reporter bio-bar-coded AuNPs labelled with PbS or CdS nanoparticles were adopted for the construction of this platform. The target proteins can be quantitatively analyzed by dissolution of the corresponding metal sulfide nanoparticles using anodic stripping voltammetry (ASV). The proposed method offers excellent selectivity and is suitable for real sample analysis. Another extension of the methods based on QDs involves the use of nanoscale metal–organic frameworks (NMOFs). Owing to the large surface area, controllable pore structure, and various functional groups, these materials provide a potential platform for immobilizing various metal ions. An electrochemical aptasensor for the simultaneous detection of oxytetracycline (OTC) and kanamycin (KAN) was recently reported [53]. It was based on Pb^2+^ or Cd^2+^ doped MOFs and RECJf exonuclease–catalyzed target recycling amplification. The striping peaks were observed at −0.55 V (Pb) and −0.84 V (Cd), which increased with the concentration of OTC and KAN, enabling their determination with the limits of detection (LODs) of 0.18 pM and 0.15 pM, respectively [53]. A similar approach, but using a circular strand-replacement DNA polymerization (CSRP) target-triggered amplification strategy instead of RECJf, was proposed for the very sensitive detection of chloramphenicol (CAP) and oxytetracycline (OTC) with LODs of 33 fM and 48 fM, respectively [56]. The nanotracers, based on high-capacity magnetic hollow porous nanoparticles (MHPs) loading Cd and Pb ions, with coupling exonuclease-assisted cascade target recycling amplification, were reported for simultaneous detection of chloramphenicol (CAP) and oxytetracycline (OTC) with detection limits of 0.15 and 0.10 ng/mL for CAP and OTC, respectively [55]. In another paper, the authors reported on the development of electrochemical aptasensors based on graphitized multi-walled carbon nanotubes (MWCNTGr) and carbon nanofibers decorated with gold nanoparticles (CNFs-AuNPs) as an amplification strategy, and Cd^2+^ and Pb^2+^ as the signal tracers for simultaneous detection of kanamycin (KAN) and streptomycin (STR), with LODs of 74.50 pM for KAN and 36.45 pM for STR. The use of MWCNTGr and CNFs-AuNP composites resulted in a 4.6-fold signal amplification for the aptasensor [60]. A simultaneous electrochemical detection of three antibiotics, streptomycin (STR), chloramphenicol (CHL), and tetracycline (TET), based on aptamers and quantum dots, PbS, CdS, and ZnS, was achieved with nanomolar-level detection limits [54]. Recently, a multiplexed electrochemical aptasensor for kanamycin (KANA) and ampicillin (AMP) detection, using Cd^2+^ and Pb^2+^-encoded apoferritin probes and double stirring bars–assisted target recycling for signal amplification, was reported. The detection limits in fM range, 18 fM for KANA and 15 fM for AMD, were reported [64].

**Figure 1:**
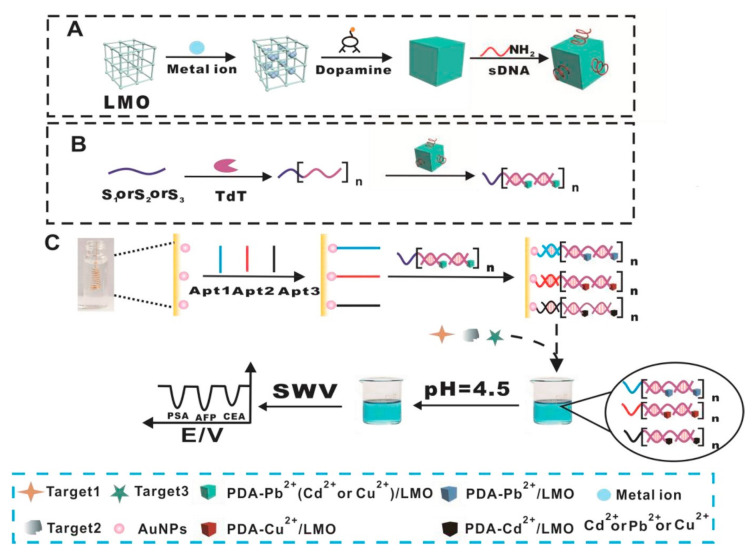
(**A**) The procedure of an encoded labels fabrication, (**B**) scheme of the DNA-(LMO-M) encoded probes formation, and (**C**) the multiplex detection scheme of serum tumor markers (reprinted with permission from Wang et al. 2021) [65].

A new type of inorganic mixed oxide, LaMnO_3_, with a perovskite crystal structure was recently employed for designing an assay for simultaneous detection of multiple tumor markers, such as the alpha fetoprotein (AFP), carcinoembryonic antigen (CEA), and prostate specific antigen (PSA) [65]. In this work, polydopamine (PDA) sealed LaMnO_3_ nanoparticles (LMO) were used for the immobilization of metal ions, Pb^2+^, Cd^2+^, and Cu^2+^, and on specific aptamers on their outer surfaces, leading to the formation of encoded probes, as shown in Figure 1 (reprinted from ref. [65]). Upon incubation with specific tumor markers, the encoded probes were released using a competitive replacement strategy. A square wave voltammetry was used for the detection of metals Pb^2+^, Cd^2+^, and Cu^2+^, with concentrations proportional to the corresponding markers: AFP, CEA, and PSA. Multiplex signal amplification was achieved by the use of stirring rods decorated with many encoded probes with large numbers of bound metal ions.

## 3. Redox-Probe Based Electrochemical Aptasensors for Simultaneous Detection of Multiple Analytes

**Scheme 3:**
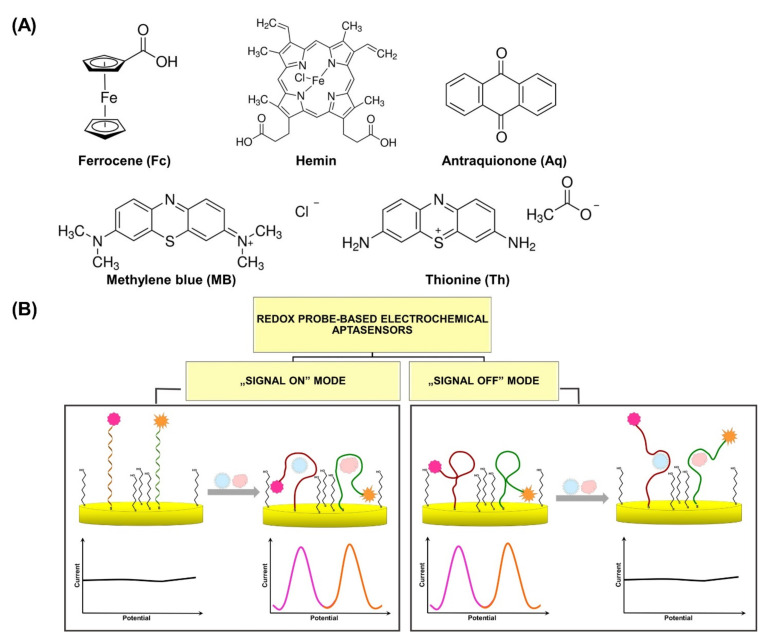
(**A**) Chemical structures of typical electroactive labels widely used for aptamer bioconjugation, and (**B**) general scheme of redox probe-based electrochemical aptasensors working principles.

Among the different redox signal tags, the compounds most frequently used in constructing electrochemical biosensors include ferrocene (Fc), anthraquinone (AQ), thionine (Th), methylene blue (MB), and hemin [66,67,68,69] (Scheme 3A). These redox compounds offer promising alternatives to heavy metal ions, mainly because of their greater environmental friendliness and lower toxicity to biological dyes [70]. A redox tag, properly selected for an application in an electrochemical biosensor, must exhibit high reversibility of its redox processes, and the redox potential should be located in the appropriate potential window depending on the composition of the working electrode material. The selected redox compound should also show high stability and the ability to bind to nanoparticles or to form conjugates with the applicable receptor molecule without affecting its recognition functionality [66,71]. From among the aptasensors proposed for simultaneous multiple analytes detection, two types of sensors have most often been considered:

(1) Structure-switching sensors [72], in which the redox-tag labelled aptamer alters the conformation upon binding a target molecule, resulting in changes in the rate of electron transfer between the redox tag and electrode surface [73,74,75,76,77,78,79,80,81,82,83], and
(2) Sensors based on bioconjugates containing nanomaterials, such as gold nanoparticles (AuNPs), gold nanorods (AuNRs), or nano zirconium-metal organic framework (NMOF), modified further with aptamer and redox tag molecules [70,84,85,86].

Depending on the sensor architecture, different signaling mechanisms are possible. In both sensor types, changes in the redox peak current of the electroactive tag are recorded and correlated with the analyte concentration changes. Such systems are often referred to as working in the “signal on” or “signal off” mode (Scheme 3B), meaning that the analytical signal either increases or decreases with increasing analyte concentrations.

Originally, the concept of an aptamer-based electrochemical sensor (AB-ES) was preceded by the design of an electrochemical DNA sensor (DNA-ES). The idea of a DNA-ES evolved from the applications of oligonucleotide probes modified with redox probes for covalent attachment to the surface of an electrode. The changes in the efficiency of electron transfer between the electrode and redox label underlie the principles of the mechanism of signal generation [87]. Selected examples of redox-probe-based multiplex electrochemical aptasensors are presented in Table 2.

**Figure 2:**
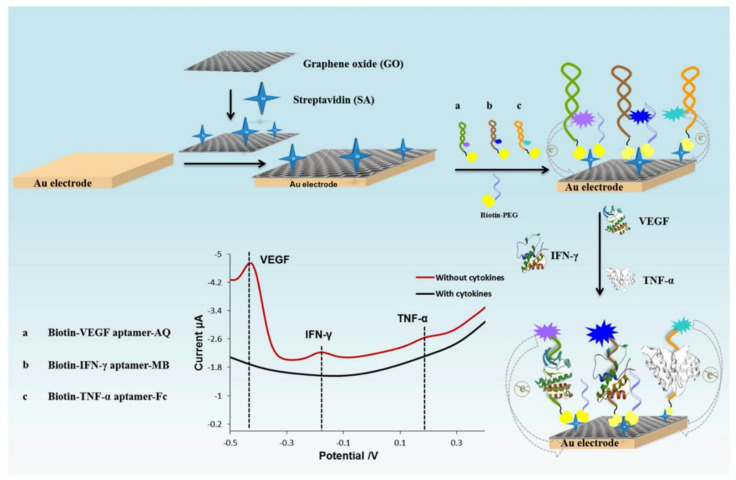
The scheme of simultaneous determination of VEGF, IFN-γ, and TNF-α using an electrochemical aptasensor labelled with redox markers aptamers (reprinted with permission from Shen et al. 2021 [83]).

In the latest example of multiplex electrochemical sensors with structure-switching aptamers, the electrochemical detection of three cytokines, VEGF, IFN-γ, and TNF-α, by three aptamers tagged with AQ, MB, and Fc, respectively, was proposed for the first time [83]. The sensor design and typical electrochemical characteristics are presented in Figure 2 (reprinted from ref. [83]). In the proposed multiplex aptasensor, a composite consisting of a graphene oxide (GO) and streptavidin (SA) was immobilized on the surface of a gold electrode by means of physical adsorption, thus providing a large loading area for the incorporation of biotinylated aptamers and biotinylated polyethylene glycol. The authors claimed this platform has high stability and anti-fouling capabilities and were able to successfully apply this multiplex aptasensor for simultaneous and real-time detection of three cytokines in human serum and artificial sweat. However, because of the weak nature of the physical adsorption used to immobilize GO/SA basal film on a gold electrode surface, the proposed multiplex aptasensor has very limited long-term stability. It is also likely that in a biological medium, in the presence of biothiols such as glutathione or cysteine, which have a high affinity to Au surfaces, the stability of basal films of GO/SA will be further reduced.

**Figure 3:**
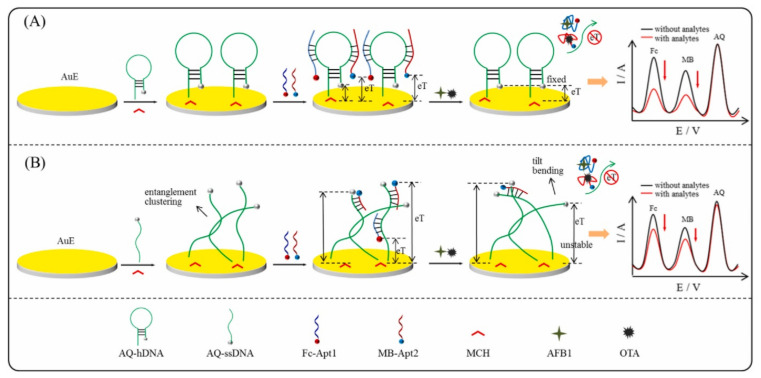
Schematic illustration of simultaneous determination of AFB1 and OTA using (**A**) the hDNA-based and (**B**) ssDNA-based dual-ratiometric electrochemical aptasensor (reprinted with permission from Zhu et al. [82]).

Multiplex electrochemical aptasensors have recently been applied for the detection of common toxins in food samples. A novel ratiometric electrochemical aptasensor has been reported for simultaneous detection of aflatoxin B1 (AFB1) and ochratoxin A (OTA) [82]. In the construction of the sensing layer in this multiplex sensor (Figure 3, reprinted from ref. [82]), a hairpin DNA (hDNA) labelled with anthraquinone (AQ), an AFB1 aptamer labelled with Fc (Fc-Apt1), and an OTA aptamer labelled with methylene blue (MB-Apt2) were employed. In this multiplex sensor, the hDNA used was coded to exhibit specific binding sites to capture both Fc-Apt1 and MB-Apt2. The detection mechanism of this aptasensor was based on the dissociation of Fc-Apt1 and MB-Apt2 upon recognition of targets that caused a decrease of the oxidation current of Fc (IFc) and MB (IMB), while no changes in the oxidation current for AQ were observed. Thus, the ratiometric signals IFc/IAQ and IMB/IAQ could be used to quantify AFB1 and OTA.

**Figure 4:**
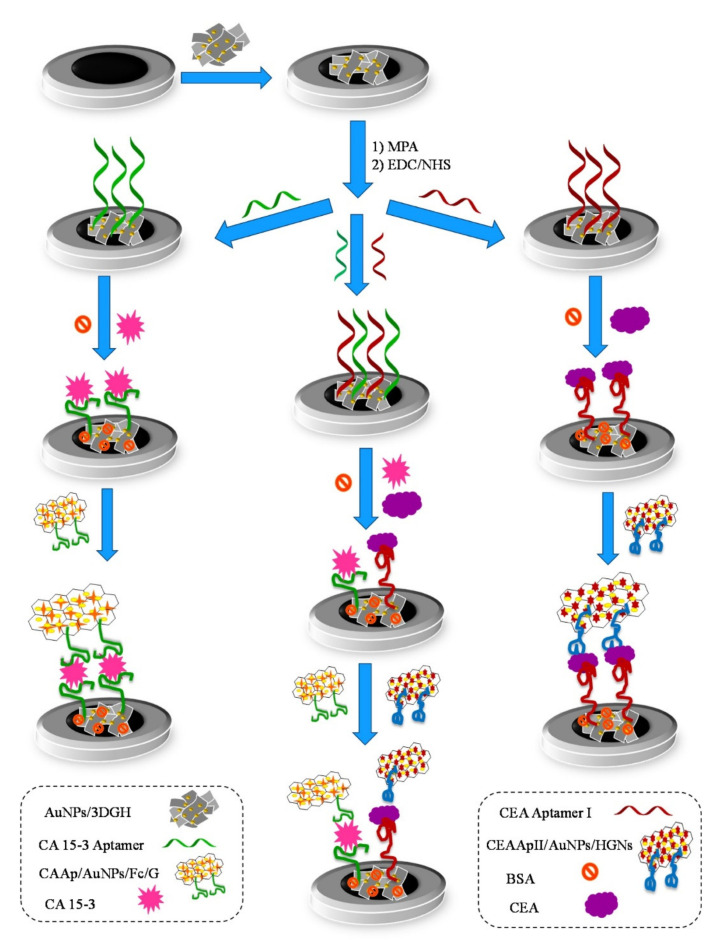
The scheme of the sandwich–type aptasensor preparation steps for the single and simultaneous detection of CEA and CA 15–3 biomarkers (reprinted with permission from Shekari et al. [86]).

A new type of multiplex sensor based on bioconjugates containing nanomaterials, modified with aptamers and redox tag molecules, was developed by Shekari et al. [86]. In these sensors, gold nanoparticles were embedded in a three-dimensional graphene hydrogel (AuNPs/3DGH) and deposited onto a glassy carbon electrode, as depicted in Figure 4 (reprinted from ref. [86]). To this basal film, carcinoembryonic antigen (CEA) aptamer and cancer antigen 15-3 (CA 15-3) aptamer were bonded using EDC/NHS chemistry. Prepared in this way, the sensory films were able to bind CEA and CA 15-3 cancer biomarkers. After adding secondary aptamers tagged with AuNPs and redox labels (Fc and hemin, respectively), the bound target analytes, CEA and CA 15-3, could be determined with high sensitivity. Here, the introduction of nanomaterials allowed a large surface area to be obtained, as well as an increase in the rate of electron transfer and electrical conductivity.

Further improvement in the electron transfer rate for the redox processes was achieved by Han et al. [84] by using co-reduced molybdenum disulphide and gold nanoparticles (rMoS2-Au) as the platform for simultaneous determination of zearaleone and fumonisin B1. Owing to the features of rMoS2-Au, such as superior electron transfer rates, large surface areas, and the ability to tightly bind large numbers of aptamer molecules, the aptasensors developed have shown high sensitivity, excellent selectivity, and good stability.

Recently, Wei and co-workers designed multiplex aptasensors with functionalized gold nanorods (AuNRs) playing the roles of both the recognition elements and signal amplification elements [85]. Two groups of nanorods were synthesized: (1) Apt1-AuNRs-Th, where AuNRs were functionalized with immobilized thionine (Th) and thiolated ochratoxin A aptamer (Apt1), and (2) Apt2-AuNRs-Fc, where AuNRs were functionalized with thiolated ferrocene (Fc) and thiolated fumonisin B1 aptamer (Apt2). Both bioconjugates, Apt1-AuNRs-Th and Apt2-AuNRs-Fc, were able to simultaneously hybridize with complementary cDNA present on the surface of a gold electrode, forming unique Y-shaped DNA structures. This, in consequence, led to the generation of two electrochemical signals for the reduction/oxidation of thionine and ferrocene, respectively.

Upon the binding of aptamers with related target toxins, the release of bioconjugates and the corresponding decrease in peak current densities occurred. Thus, the latter can be correlated with a change in concentration of the respective analytes. Similarly, as in the case of metals-based electrochemical aptasensors, hierarchically porous nano-metal organic frameworks functionalized with amino groups (HP-MOFs-NH_2_) have been used for the encapsulation of small redox organic molecular agents, such as methylene blue (MB) and ferrocene (Fc). Prepared in this way, HP-MOFs-NH_2_ frameworks have been decorated with amino-functionalized DNA aptamers via glutaraldehyde coupling and selected as good candidates for simultaneous detection of kanamycin (KANA) and chloramphenicol (CAP), respectively [70].

## 4. Enzyme-Based Electrochemical Aptasensors for Simultaneous Multiple Analytes Detection

**Scheme 4:**
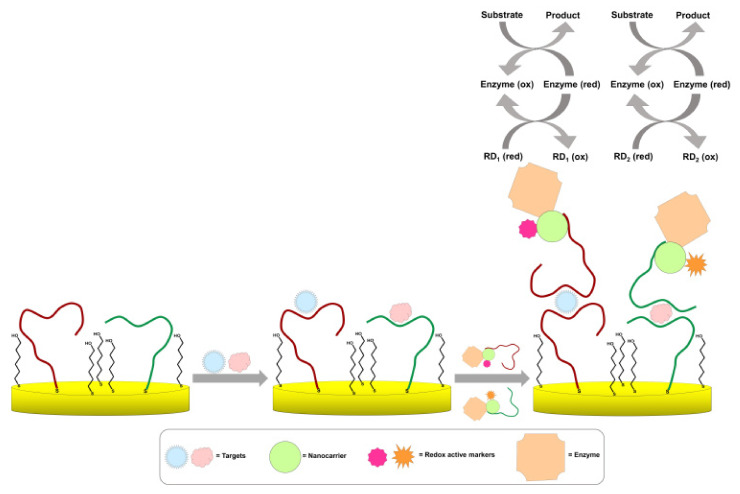
Schematic illustration of enzyme-driven multiplex aptasensing process.

Enzyme labels have been used as signal amplifiers in electrochemical biosensors for many years [89]. However, the use of enzymatic labels or modern nanomaterials is not sufficient to attain ultra-high sensitivity in biosensors. Therefore, a new approach based on combining an enzymatic reaction with redox cycling or the application of multienzyme labels on each detection probe was introduced [90]. The reactions involved in an enzyme-driven multiplex aptasensing process are illustrated in Scheme 4 and examples of enzyme-based multiplex electrochemical aptasensors are presented in Table 3.

**Figure 5:**
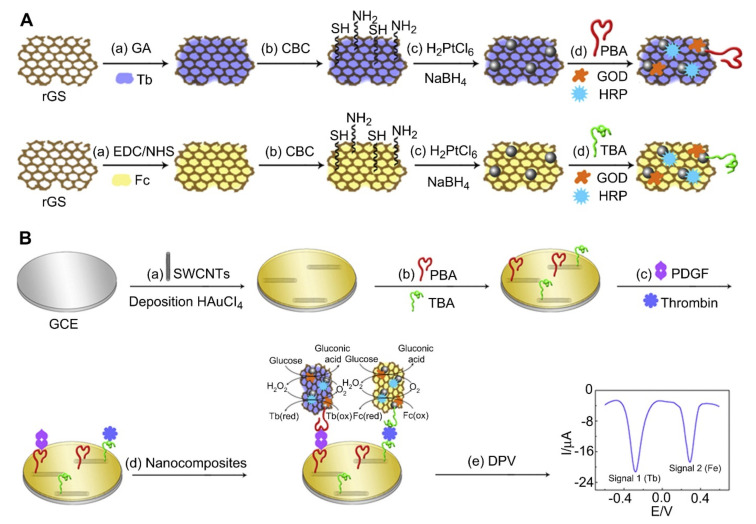
(**A**) The illustration of nanocomposites preparation steps based on aptamers, GOD, and HRP multi-labeled PtNPs-redox probes-rGS, and (**B**) the fabrication process of the stepwise aptasensor and differential pulse voltammogram of current response toward PDGF and thrombin (reprinted with permission from Bai et al. [91]).

From among many enzymes, the oxidoreductases, including glucose oxidase (GOx), choline oxidase (ChOD), and horseradish peroxidase (HRP), are the most frequently applied enzymes when designing electrochemical biosensors [89]. As an example of an enzyme-based multiplex aptasensor system, a sandwich-type electrochemical aptasensor for simultaneous detection of platelet-derived growth factor (PDGF) and thrombin, with LODs in the pM range, can be considered [91]. The proposed sensing mechanism is illustrated in Figure 5 (reprinted from ref. [91]). In this case, single-walled carbon nanotubes decorated with gold nanoparticles (AuNPs@SWCNTs) were used to capture primary aptamers (Apts I): PDGF-binding aptamers (PBA) and thrombin-binding aptamers (TBA). Nanocomposites containing platinum nanoparticles (PtNPs) and reduced graphene oxide sheets (rGS), modified with redox probes toluidine blue (Tb) and ferrocene (Fc), were applied as the carriers for secondary aptamers (Apts II) and bienzyme, glucose oxidase (GOx) and horseradish peroxidase (HRP). After a complex formation between the corresponding aptamers, the platelet-derived growth factor (PDGF), and thrombin is achieved, the GO nanosheets catalyze the reduction of glucose leading to H_2_O_2_ generation. Next, the reduction of H_2_O_2_ by HRP and PtNPs with the aid of Tb and Fc takes place.

**Figure 6:**
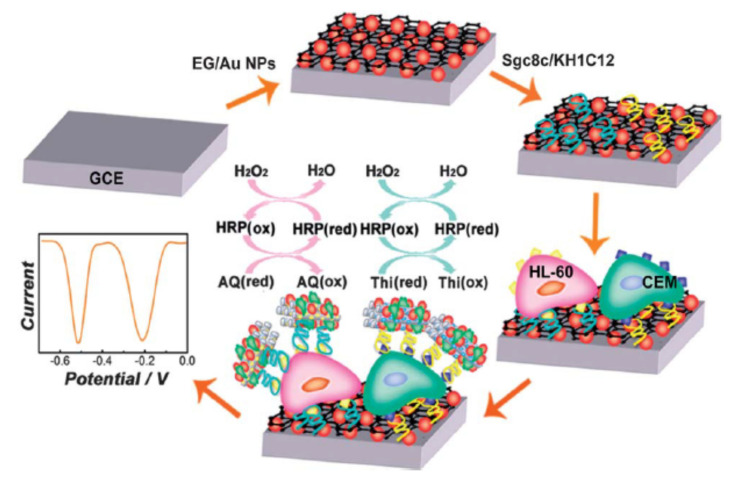
An illustration of the major steps of electrochemical cytosensor construction (reprinted with permission from Zheng et al. [92]).

The electrochemical cytosensing platform for simultaneous detection of acute myeloid leukemia (AML) and acute lymphocytic leukemia (ALL) cells is another example of an enzyme-catalyzed signal-amplification-based aptasensor [92]. The major steps in this electrochemical cytosensor fabrication, depicted in Figure 6 (reprinted from ref. [92]), are based on (1) modification of glassy carbon electrodes with a multi-layer AuNP-graphene composite film, (2) assembly of thiol-terminated cells targeting aptamers (SH-KH1C12 and SH-Sgc8c), (3) the capturing of specific cells, HL-60 and CEM, and (4) recognition of both cell types by the corresponding aptamers (HL-60 cells by KH1C12 and CEM cells by Sgc8c), immobilized on the mesoporous nanostructure of silica (SBA-15) together with HRP, anthraquinone (AQ), and thionine (Th). In this way, a super-structured sandwich–type ultrasensitive cytosensing approach was developed.

## 5. Future Perspectives of Multiplex Electrochemical Aptasensors

Based on the evaluation of the most recently proposed electrochemical aptasensors for simultaneous detection of multiple analytes, we can see that this research area is being extensively development. There is no doubt that the electrochemical aptasensors reported so far offer high efficiency for multiplex analyte determination. However, opportunities for further improvement remain. The following assessment of the progress, as well as the problems with the pitfalls encountered, is related to the specific features and phenomena of multiplex electrochemical aptasensors and their analytical signal amplification mechanisms.

First, the most frequently used nanomaterials are porous inorganic materials, such as metal organic frameworks (MOFs) or mesoporous silica particles. They offer such characteristic features as a large specific surface area and a capability for high loading density of biorecognition molecules. However, the insufficient encapsulation of metal ions or high background caused by metal ion leakage are the main drawbacks of metal ion probe-based electrochemical aptasensors. Moreover, the elution of the metal ions from the probes has been achieved using HNO_3_ or HCl. These harsh conditions not only cause pollution to the environment but can also be dangerous to the personnel conducting the experiments. Finding a solution to avoid these problems is the key challenge for these aptasensors. Therefore, the development of new materials characterized by a large specific surface area available for the adsorption of metal ions, which could be readily removed by medium exchange, is highly desirable.

Second, the use of toxic heavy metal ions as signal tracers and the highly complicated procedures for aptasensor fabrication are disadvantages that somewhat reduce their practical significance. However, there is still a chance to design systems with simple configurations and straightforward operation. Furthermore, attractive alternative redox labels for future electrochemical biosensors are highly sought-after. These new candidates should display even better characteristics than those shown by already known redox compounds, in terms of diffusion coefficients, the heterogeneous electron transfer rate constant, current density, and peak separation. Moreover, the possible alternative compounds should have the ability to be easily conjugated to biomolecules or nanomaterials.

Third, taking into account the limited number of multiplex enzyme-based electrochemical aptasensors, there is still room for designing new sensors, especially since the redox processes catalyzed by enzymes could significantly amplify the amperometric signals as compared to the direct amperometric signals from redox reporters, leading to an improvement in the sensitivity of the sensor. The testing of new nanomaterials in this branch is expected, not only for the purpose of accelerating electron transfer, but also to facilitate the immobilization of enzyme molecules.

## Figures and Tables

**Table 1:** Multiplex electrochemical aptasensors based on quantum dots, metal compound semiconductor NPs, and metal ions.

Analytes	Method	LOD/Range	Signal Tags	Ref.
thrombin, lysozyme	SWSV	both analytes: 20 ngL^−1^/20–500 ng L^−1^	QDs nanocrystal: CdS, PbS	[48]
adenosine, thrombin	ASV	adenosine: 8.8 × 10^−12^ M/1 × 10^−11^–2.0 × 10^−9^ M, thrombin: 7.6 × 10^−13^ M/1.0 × 10^−12^–3.0 × 10^−10^ M	CdS, PbS	[49]
ATP, cocaine	SWV	ATP: 30 nM, cocaine: 50 nM	CdS, PbS	[50]
CAP, PCB-72	SWV	CAP, PCB-72: 0.3 pgmL^−1^/0.001–100 ngmL^−1^	Cd^2+^ and Pb^2+^ encoded MSPEIs	[51]
CAP, PCB-72	SWSV	CAP: 0.33 pg mL^−1^, PCB72: 0.35 pg mL^−1^/0.001–100 ng mL^−1^	CdS, PbS	[52]
OTC, KANA	SWV	OTC: 0.18 pM, KANA: 0.15 pM/0.5 pM–50 nM	NMOF labelled Pb^2+^ or Cd^2+^	[53]
STR, CAP, TET	SWASV	STR: 10 nM, CHL: 5 nM, TET: 20 nM	PbS, CdS, ZnS QDs	[54]
CAP, OTC	SWV	CAP: 0.15 ngmL^−1^, OTC: 0.10 ngmL^−1^/0.0005ng–50 ngmL^−1^	Cd^2+^ or Pb^2+^ encoded MHPs	[55]
KANA, CAP	SWV	KANA: 0.16 pM, CAP: 0.19 pM	NMOF labelled Pb^2+^ or Cd^2+^	[56]
CAP, OTC	SWV	CAP: 33 fM, OTC: 48 fM	NMOF labelled Pb^2+^ or Cd^2+^	[57]
OTA, FB1	SWV	OTA: 5 pgmL^−1^, FB1: 20 pgmL^−1^ OTA: 10 pgmL^−1^–10 ngmL^−1^, FB1: 50 pgmL^−1^–50 ngmL^−1^	CdTe or PbS QDs coated silica	[58]
CEA, mucin-1	DPV	both analytes: 3.3 fM/0.01 pM-100 nM	Au/BSA nanospheres loading Pb^2+^, Cd^2+^	[59]
KANA, STR	DPV	KANA: 74.50 pM, STR: 36.45 pM/0.1–100 nM	CdS, PbS	[60]
CEA, AFP	DPV	CEA: 1.8 pg mL^−1^, AFP: 0.3 pg mL^−1^	Au@Cu_2_O and Au@Ag	[61]
miRNA 155, miRNA 122	DPV	miRNA 155: 6.7 fM, miRNA 122: 1.5 fM/0.01–1000 pM	AgNCs@AuNPs:Cu_2_O@AuNPs	[62]
haemophilia A-related microRNAs:miR-1246 and miR-4521	DPV	miR-1246: 0.19 fM, miR-4521: 0.28 fM/1 fM–1 µM	QDs@ZIF-8: PbS@ZIF-8 and CdS@ZIF-8	[63]
KANA, AMP	SWV	KANA: 18 fM, AMP: 15 fM/0.05 pM–50 nM	apoferritin loading Cd^2+^, Pb^2+^	[64]
AFP, CEA, PSA	SWV	CEA: 3.6 × 10^−4^ ng mL^−1^, AFP: 3,4 × 10^−4^ ng mL^−1^, PSA: 2.8 × 10^−4^ ng mL^−1^	PDA-Pb^2+^, Cd^2+^, Cu^2+^-LMO	[42]

AFP, alpha-fetoprotein; AgNCs@AuNPs, Au@Ag, Au@Cu2O, Cu2O@AuNPs, shell encoded gold nanoparticles; AMP, ampicillin; ASV, anodic stripping voltammetry; ATP, adenosine triphosphate; Au, gold; AuNPs, gold nanoparticles; BSA, bovine serum albumin; CAP, chloramphenicol; CEA, carcinoembryonic antigen; DPV, differential pulse voltammetry; FB1, fumonisin B1; KANA, kanamycin; LMO, LaMO3 perovskite; MHPs, magnetic hollow porous nanoparticles; miR-1246 and miR-4521, haemophilia A-related microRNAs; MSPEIs, nanospherical branched polyethylene imine brushes; NMOF, nanoscale metal organic framework; OTA, ochratoxin A; OTC, oxytetracycline; PCB-72, polychlorinated biphenyl-72; PDA, polydopamine; PSA, prostate specific antigen; QDs, quantum dots; QDs@ZIF-8, heavy metals quantum dots-encapsulated metal-organic frameworks; STR, streptomycin; SWASV, square wave anodic stripping voltammetry; SWSV, square-wave stripping voltammetry; SWV, square wave voltammetry; TET, tetracycline; and ZIF-8, zeolitic imidazolate framework.

**Table 2:** Multiplex electrochemical aptasensors based on redox signal tags.

Analytes	Method	LOD/Range	Signal Tags	Ref.
ATP, cocaine	SWV	ATP: 0.1 pM, cocaine: 1.5 pM	Th, Fc	[73]
MUC-1, VEGF_165_	SWV	0.33 nM/1 nM–20 nM	Fc	[75]
TB, OTA	DPV	TB: 0.05 pM, OTA: 0.12 pM/TB: 0.1 pM–40 nM, OTA: 0.4 pM–35 nM	AQ, hemin	[76]
Ang, Tob	SWV	Ang: 0.07 pM, Tob: 20 fM/Ang: 0.2 pM–10 nM, Tob: 50 fM-5 nM	MB, Fc	[77]
IFN-γ, TNF-α	SWV	IFN-γ: 6.35 ngmL^−1^, TNF-α: 5.46 ngmL^−1^	AQ, MB	[79]
IFN-γ, Lys	SWV	IFN-γ: 1.14 × 10^−3^ nM, Lys: 0.0164 nM/IFN-γ: 0.01–10 nM, Lys: 0.1–100 nM	MB, Fc	[78]
MUC-1, CEA	SWV	MUC-1: 0.6 nM, CEA: 2.75 ngmL^−1/^MUC-1: 10 nM–100 nM, CEA: 30 ngmL^−1^–300 ngmL^−1^	MB	[80]
Ag^+^, Hg^2+^	DPV	Ag^+^: 2 pM, Hg^2+^: 7.5 pM/Ag^+^: 0.01 nM–5µM, Hg^2+^: 0.5 nM–50 µM	AQ, Fc	[81]
KANA, CAP	SWV	KANA: 35 fM, CAP: 21 fM/KANA, CAP: 1 × 10^−4^–50 nM	MB, Fc	[70]
ZEN, FB1	DPV	ZEN, FB1: 5 × 10^−4^ ngmL^−1/^ZEN: 1 × 10^−3^–10 ngmL^−1^, FB1: 1 × 10^−3^–1 × 10^2^ ngmL^−1^	Th, Fc	[84]
OTA, FB1	DPV	OTA: 0.47 pgmL^−1^, FB1: 0.26 pgmL^−1/^1.0 pgmL^−1^–100 ngmL^−1^	Th, Fc	[85]
AFB1, OTA	ACV	AFB1: 4.3 pgmL^−1^, OTA: 13.3 pgmL^−1/^AFB1: 10–3000 pgmL^−1^, OTA: 30–10,000 pgmL^−1^	Fc, MB	[82]
CEA, CA 15-3	DPV	CEA: 11.2 pg mL^−1^, CA 15-3 132 × 10^−2^ U mL^−1^/CEA: 5.0 × 10^−2^–60.0 ng mL^−1^, CA 15-3: 5.0 × 10^−2^–100.0 U mL^−1^	Hemin, Fc	[86]
VEGF, IFN-γ, TNF-α	SWV	5 pg mL^−1^ each cytokine/VEGF: 5–300 pg mL^−1^, IFN-γ: 5–300 pg mL^−1^, TNF-α: 5–200 pg mL^−1^	AQ, MB, Fc	[83]
PSA, sarcosine	SWV	PSA: 2.5 fg mL^−1^, sarcosine: 14.4 fg mL^−1^	MB, Fc	[88]

ACV, alternating current voltammetry; AFB1, aflatoxin B1; Ang, angiogenin; AQ, antraquinone; ATP, adenosine triphosphate; CA 15-3, carbohydrate antigen 15-3; CAP, chloramphenicol; CEA, carcinoembryonic antigen; DPV, differential pulse voltammetry; FB1, fumonisin B1; Fc, ferrocene; IFN-γ, interferon gamma; KANA, kanamycin; Lys, lysozyme; MB, methylene blue; MUC-1, human mucin-1; OTA, ochratoxin; PSA, prostate specific antigen; SWV, square wave voltammetry; TB, thrombin; Th, thionine; TNF-α, tumor necrosis factor alpha; Tob, thrombin; VEGF, vascular endothelial growth factor; and ZEN, zearalenone.

**Table 3:** Multiplex electrochemical aptasensors based on enzymatic tags.

Analytes	Method	LOD/Range	Signal Tags	Ref.
PDGF, thrombin	DPV	PDGF: 8 pM/0.01–35 nM Thrombin: 11pM/0.02–45 nM	bienzyme: glucose oxidase, HRP, Tb, Fc	[91]
AML, ALL	DPV	~350 cells per mL/5 × 10^2^–1 × 10^7^ cells per mL	HRP, Aq, Thi	[92]

ALL, acute lymphocytic leukemia cells; AML, acute myeloid leukemia cells; Aq, anthraquinone; DPV, differential pulse voltammetry; Fc, ferrocene; HRP, horseradish peroxidase; PDGF, platelet-derived growth factor; Tb, toluidine blue; and Thi, thionine.

## Data Availability

Not applicable.
